# Fragranced consumer products: exposures and effects from emissions

**DOI:** 10.1007/s11869-016-0442-z

**Published:** 2016-10-20

**Authors:** Anne Steinemann

**Affiliations:** 1 Department of Infrastructure Engineering, Melbourne School of Engineering, The University of Melbourne, Melbourne, VIC 3010 Australia; 2 College of Science, Technology and Engineering, James Cook University, Townsville, QLD 4811 Australia; 3 Climate, Atmospheric Sciences, and Physical Oceanography, Scripps Institution of Oceanography, University of California, San Diego, La Jolla, CA 92093 USA

**Keywords:** Fragrance, Consumer product, Indoor air quality

## Abstract

Fragranced consumer products, such as cleaning supplies, air fresheners, and personal care products, are a primary source of indoor air pollutants and personal exposure. Previous research indicates that fragranced products can trigger adverse health effects, with implications for workplaces and public places. This is the first study to examine the multiple dimensions of exposures related to fragranced products and effects in the US population. The study investigated the prevalence and types of fragranced product exposures, associated health effects, awareness of product emissions, and preferences for fragrance-free policies and environments. Data were collected using an online survey with a nationally representative population (*n* = 1136) of adults in the USA. Overall, 34.7 % of the population reported health problems, such as migraine headaches and respiratory difficulties, when exposed to fragranced products. Further, 15.1 % have lost workdays or a job due to fragranced product exposure in the workplace. Also, 20.2 % would enter a business but then leave as quickly as possible if they smell air fresheners or some fragranced product. Over 50 % of the population would prefer that workplaces, health care facilities and professionals, hotels, and airplanes were fragrance-free. While prior research found that common fragranced products, even those called green and organic, emitted hazardous air pollutants, more than two thirds of the population were not aware of this, and over 60 % would not continue to use a fragranced product if they knew it emitted such pollutants. Results from this study provide strong evidence that fragranced products can trigger adverse health effects in the general population. The study also indicates that reducing exposure to fragranced products, such as through fragrance-free policies, can provide cost-effective and relatively simple ways to reduce risks and improve air quality and health.

**Electronic supplementary material:**

The online version of this article (doi:10.1007/s11869-016-0442-z) contains supplementary material, which is available to authorized users.

## Introduction

Society is suffused with fragranced consumer products: air fresheners, cleaning products, soaps, hand sanitizers, laundry supplies, and personal care products, to name a few out of hundreds.^1^ Fragranced products emit a range of volatile organic compounds (VOCs), such as terpenes (e.g., limonene), which often dominate pollutants found indoors, and generate secondary pollutants such as formaldehyde (e.g., Nazaroff and Weschler 2004).

Fragranced products have been associated with a range of adverse health effects, such as migraine headaches, asthma attacks, respiratory difficulties, neurological problems, mucosal symptoms, and contact dermatitis (Kim et al. 2015; Elberling et al. 2005; Millqvist and Löwhagen 1996; Kumar et al. 1995; Kelman 2004; Caress and Steinemann 2004, 2005; Johansen 2003; Rastogi et al. 2007; Sealey et al. 2015). In two previous national surveys of the US population, 19 % reported breathing difficulties, headaches, or other health problems when exposed to air fresheners and deodorizers, and 10.9 % reported health problems from the scent of laundry products vented outdoors (Caress and Steinemann 2009).

Despite numerous laws designed to protect human health and the environment, no law in the US requires the disclosure of all ingredients in fragranced consumer products (Steinemann 2009). Protections on ingredient disclosure depend on the product. First, for all fragranced consumer products, the general term “fragrance” can be listed on the label, or a related term (such as “perfume”), rather than the specific ingredients in a fragrance. Yet an individual “fragrance” in a product is typically a complex mixture of several dozen to several hundred chemicals (Bickers et al. 2003), primarily synthetic compounds (Somogyi et al. 1998), among nearly 3000 compounds documented as fragrance ingredients (IFRA 2011). Second, for products such as air fresheners, laundry supplies, cleaning products, and others regulated by the US Consumer Product Safety Commission, ingredients do not need to be fully listed on either the product label or the material safety data sheet (MSDS). Also, these products do not need to list the presence of a “fragrance” on either the label or MSDS. Third, for products such as personal care products, cosmetics, and others regulated by the US Food and Drug Administration, ingredients need to be listed on the product label, but not on the MSDS. The general term “fragrance” can be listed on the label instead of the specific ingredients in the fragrance, but the fragrance term or ingredients do not need to be listed on the MSDS.

Previous studies that analyzed fragranced product emissions found that relatively few ingredients were disclosed to the public (e.g., Uhde and Schulz 2015; Steinemann et al. 2011). For instance, Steinemann (2015) found more than 156 VOCs emitted from 37 fragranced consumer products, the most common being limonene, alpha-pinene, beta-pinene, and other terpenes. Of these 156 VOCs, 42 VOCs were classified as toxic or hazardous under US federal laws, and each product emitted at least one of these chemicals. Moreover, emissions of carcinogenic hazardous air pollutants from so-called green or organic fragranced products were not significantly different from regular fragranced products. However, of over 550 volatile ingredients emitted collectively, and over 230 classified as toxic or hazardous, fewer than 3 % were disclosed on the product labels, MSDSs, or websites.

Product emissions can affect not only indoor environments but also outdoor air quality. For instance, emissions from dryer vents, during the use of fragranced laundry products, contain numerous VOCs that affect outdoor air quality, such as acetaldehyde, a hazardous air pollutant (Steinemann et al. 2011). While these pollutants are regulated from outdoor sources, they are unregulated when coming from indoor sources (such as laundry dryer vents), even though emissions can affect outdoor air quality and human health.

Individuals report health problems when exposed to fragranced products in society, other than through intentional use of products. Secondhand scents (as termed in this article) refers to indirect or involuntary exposure to fragranced products (in an analogy to secondhand smoke). As a response, fragrance-free policies (similar to smoke-free policies) have been implemented by businesses, agencies, and institutions in the USA and other countries (e.g., CDCP 2009; CCOHS 2015) to restrict the use of fragranced products within indoor environments such as workplaces, schools, hospitals, and public places.

This article reports results from the first national population survey to investigate a range of exposures and effects associated with fragranced product emissions, preferences for fragrance-free environments, and implications for air quality, indoor environments, and health. Contributions include new data and insights on the extent and impacts of the problem and pathways for solutions.

## Methods

To investigate the prevalence and types of exposures, health effects, and societal impacts, an on-line survey was conducted of the adult American population, using a national random sample representative of age, gender, and region (*n* = 1136, confidence limit = 95 %, confidence interval = 3 %). The survey instrument was developed and tested over a 2-year period, including cognitive testing with 10 individuals and piloting with over 100 individuals, before full implementation in June 2016. The survey drew upon participants from a large web-based USA panel (over 5,000,000 people) held by Survey Sampling International. Participant recruitment followed a randomized process as detailed in SSI (2016). All responses were anonymous. Survey completion time was approximately 10 min. The survey response rate was 94 %. The research study received ethics approval from the University of Melbourne. Details on the survey methodology are provided as a supplemental document.

Survey questions investigated the following dimensions: use and exposure to fragranced products, both from one’s own use and from others’ use; health effects related to exposures to certain products and exposure settings; impacts of fragrance exposure in the workplace and in society; awareness of fragranced product ingredients and labeling; preferences for fragrance-free environments and policies; and demographic information.

Fragranced products were categorized as follows: (a) Air fresheners and deodorizers (e.g., sprays, solids, oils, disks); (b) Personal care products (e.g., soaps, hand sanitizer, lotions, deodorant, sunscreen, shampoos); (c) Cleaning supplies (e.g., all-purpose cleaners, disinfectants, and dishwashing soap); (d) Laundry products (e.g., detergents, fabric softeners, dryer sheets); (e) Household products (e.g., scented candles, toilet paper, trash bags, baby products); (f) Fragrance (e.g., perfume, cologne, after-shave); and (g) Other.

Health effects were categorized as follows: (a) Migraine headaches; (b) Asthma attacks; (c) Neurological problems (e.g., dizziness, seizures, head pain, fainting, loss of coordination); (d) Respiratory problems (e.g., difficulty breathing, coughing, shortness of breath); (e) Skin problems (e.g., rashes, hives, red skin, tingling skin, dermatitis); (f) Cognitive problems (e.g., difficulties thinking, concentrating, or remembering); (g) Mucosal symptoms (e.g., watery or red eyes, nasal congestion, sneezing); (h) Immune system problems (e.g., swollen lymph glands, fever, fatigue); (i) Gastrointestinal problems (e.g., nausea, bloating, cramping, diarrhea); (j) Cardiovascular problems (e.g., fast or irregular heartbeat, jitteriness, chest discomfort); (k) Musculoskeletal problems (e.g., muscle or joint pain, cramps, weakness); (j) Other. The categories of health effects were derived from prior surveys of fragranced products and health effects (Caress and Steinemann 2004, 2005; Miller and Prihoda 1999) and pre-tested and reviewed by a pilot group of individuals and health care professionals.

In addition to products and health effects, specific exposure contexts were investigated, which included the following: air fresheners or deodorizers used in public toilets and other places, scented laundry products vented outdoors, being in a room after it was cleaned with scented cleaning products, being near someone wearing a fragranced product, entering a business with the scent of fragranced products, fragranced soap used in public toilets, and ability to access environments that used fragranced products. Questions also investigated awareness of fragranced product emissions and ingredient disclosure, preferences for fragrance-free environments (e.g., workplaces, health care facilities, airplanes, and hotels), and lost workdays due to fragranced product exposure. Demographic questions were asked regarding age, gender, household income, and zip code in the USA.

## Results

Main findings are summarized in this section, with full results provided as supplemental documentation.

### Fragranced product use

Of the general population surveyed in America, 98.3 % are exposed to fragranced products at least once a week, from their own use: 72.8 % air fresheners and deodorizers; 88.8 % personal care products; 79.9 % cleaning supplies; 84.1 % laundry products; 77.0 % household products; 70.2 % fragrance; 3.0 % other.

Further, 92.1 % are exposed to fragranced product at least once a week, from others’ use: 57.9 % air fresheners and deodorizers; 66.1 % personal care products; 54.8 % cleaning supplies; 47.4 % laundry products; 52.3 % household products; 68.7 % fragrance; 3.2 % other.

Collectively, 99.1 % of the population are exposed to fragranced products at least once a week from their own use, others’ use, or both.

### Health effects

Overall, 34.7 % of the population reported one or more types of adverse health effects from exposure to one or more types of fragranced products. The most common types of adverse effects were as follows: 18.6 % respiratory problems; 16.2 % mucosal symptoms; 15.7 % migraine headaches; 10.6 % skin problems; 8.0 % asthma attacks; 7.2 % neurological problems; 5.8 % cognitive problems; 5.5 % gastrointestinal problems; 4.4 % cardiovascular problems; 4.0 % immune system problems; 3.8 % musculoskeletal problems; and 1.7 % other.

Of the 34.7 % of the population reporting adverse health effects, 56.1 % are female and 43.9 % are male. Thus, proportionately more females report adverse effects than males, relative to the general population (female 53.8 %, male 46.2 %).

Products and exposure situations that trigger adverse health effects include the following:

Air fresheners and deodorizers: 20.4 % reported health problems when exposed to air fresheners or deodorizers (9.5 %, respiratory problems, 7.6 % mucosal symptoms, 7.2 % migraine headaches, 5.7 % skin problems, 4.7 % asthma attacks, 3.2 % neurological problems, and others). This compares to previous studies (Caress and Steinemann 2009) that found 17.5 and 20.5 % of the population (in 2002–2003 and 2005–2006, respectively) reported headaches, breathing difficulties, or other health problems when exposed to air fresheners or deodorizers.

Scented laundry products vented outdoors: 12.5 % reported health problems from the scent of laundry products coming from a dryer vent (4.2 % mucosal symptoms, 4.0 % respiratory problems, 3.6 % skin problems, 3.3 % migraine headaches, 2.6 % gastrointestinal problems, 2.5 % asthma attacks, and others). This compares to previous studies (Caress and Steinemann 2009) that found 10.9 % of the population (in 2005–2006) reported headaches, breathing difficulties, or other health problems when exposed to the scent of laundry products vented outside.

Proximity to fragranced person: 23.6 % reported health problems from being near someone who is wearing a fragranced product (10.4 % respiratory problems, 8.6 % mucosal symptoms, 8.5 % migraine headaches, 3.9 % asthma attacks, 3.6 % neurological problems, 3.4 % skin problems, and others). This compares to previous studies (Caress and Steinemann 2009) that found 31.1 and 29.9 % of the population in (2002–2003 and 2005–2006, respectively) reported headaches, breathing difficulties, or other health problems when next to someone wearing a scented product.

Cleaning products: 19.7 % reported health problems from being in a room after it has been cleaned with scented products (9.6 %, respiratory problems, 7.3 % mucosal symptoms, 6.6 % migraine headaches, 4.1 % neurological problems, 4.0 % asthma attacks, 4.0 % skin problems, and others).

Severity of the health problems resulting from exposure to one or more types of fragranced products was investigated, using the language from the Americans with Disabilities Act (ADA 1990) to determine disability: “Do any of these health problems substantially limit one or more major life activities, such as seeing, hearing, eating, sleeping, walking, standing, lifting, bending, speaking, breathing, learning, reading, concentrating, thinking, communicating, or working, for you personally?” Of the general population, 17.2 % reported yes, indicating that the severity of effects from fragranced product exposure was potentially disabling.

### Ingredient disclosure and product claims

Fragranced products (even ones called green or organic) emit a range of volatile organic compounds, including hazardous air pollutants, but relatively few are disclosed to the public (Steinemann 2015).

Of the general population surveyed, 46.4 % were not aware that a “fragrance” in a product is typically a chemical mixture of several dozen to several hundred chemicals, and 64.6 % were not aware that fragrance chemicals do not need to be fully disclosed on the product label or material safety data sheet. Further, 67.3 % were not aware that fragranced products typically emit hazardous air pollutants such as formaldehyde, and 72.6 % were not aware that even so-called natural, green, and organic fragranced products typically emit hazardous air pollutants. However, 60.1 % would not still use a fragranced product if they knew it emitted hazardous air pollutants.

### Societal and workplace effects

Use of fragranced products in society can lead to a range of perhaps unintended yet serious consequences. Of the general population, 17.5 % are unable or reluctant to use the toilets in a public place, because of the presence of an air freshener, deodorizer, or scented product. Also, 14.1 % are unable or reluctant to wash their hands with soap in a public place, because they know or suspect that the soap is fragranced. Further, 22.7 % have been prevented from going to some place because they would be exposed to a fragranced product that would make them sick.

Significantly, 15.1 % of the general population reported that exposure to fragranced products in their work environment has caused them to become sick, lose workdays, or lose a job. Also, 20.2 % of the population reported that if they enter a business, and smell air fresheners or some fragranced product, they want to leave as quickly as possible.

Fragrance-free policies receive a strong majority of support. Of the population surveyed, 53.2 % would be supportive of a fragrance-free policy in the workplace (compared to 19.7 % that would not). Thus, 2.7 times more people would vote yes for a fragrance-free workplace than not. Also, 54.8 % would prefer that health care facilities and health care professionals be fragrance-free (compared to 22.4 % that would not). Thus, nearly 2.5 times more people would vote yes for fragrance-free health care facilities and professionals than not.

Public venues and businesses such as airplanes and hotels have been pursuing a trend of scent branding, or dispersing fragranced air through their indoor environments. However, customers may not necessarily prefer scented air.

If given a choice between flying on an airplane that pumped scented air throughout the passenger cabin, or did not pump scented air throughout the passenger cabin, 59.2 % would choose an airplane without scented air (compared to 23.6 % with scented air). Thus, over 2.5 times more passengers would prefer an airplane without scented air than with scented air. Similarly, if given a choice between staying in a hotel with fragranced air, or without fragranced air, 55.5 % would choose a hotel without fragranced air (compared to 27.8 % with fragranced air). Thus, about two times more hotel guests would choose a hotel without fragranced air than with fragranced air.

## Discussion

Fragranced product emissions can affect not only indoor air quality but also human health, workplace productivity, and societal wellbeing. Results from this study reveal that over one third of Americans suffer adverse health effects, such as respiratory difficulties and migraine headaches, from exposure to fragranced products. Of those individuals, half reported that the effects can be disabling. Yet over 99 % of Americans are exposed to fragranced products at least once a week, from their own or others’ use.

Many exposure situations are involuntary, such as air fresheners and deodorizers used in public toilets and elsewhere (20.4 % of the population reported health problems from exposure), scented laundry products vented outdoors (12.5 % reported health problems), being in a room after it was cleaned with scented products (19.7 % reported health problems), being near someone wearing a fragranced product (23.6 % reported health problems), along with workplaces, health care facilities, hotels and airplanes with fragranced air environments. A strong majority (at least twice as many individuals in support than not, in all cases surveyed) prefer that indoor environments and people would not be fragranced.

Fragranced products also restrict access in society. Of the general population, 17.5 % were unable to use toilets in public places because of air fresheners or deodorizers, 14.1 % were unable to wash their hands with soap in public places because of fragranced soap, and 22.7 % were unable to go someplace because of the presence of a fragranced product.

Moreover, fragranced product exposures have economic implications. Of those surveyed, 20.2 % would enter but then leave a business as quickly as possible if they smell fragranced products, and 15.1 % have lost workdays or a job due to fragranced product exposures in the workplace.

Importantly, adverse effects resulting from exposure to fragranced products, such as in workplaces and public places, raise concerns about liability. For instance, individuals can suffer acute health effects, such as an asthma attack, if they enter a restroom that uses air fresheners. If they are unable to access a restroom due to the presence of an air freshener, then that poses a potential violation of the Americans with Disabilities Act.

Fragranced product manufacturers are not required to disclose all ingredients in their formulations. This lack of disclosure can impede efforts to understand and reduce adverse effects associated with potentially harmful compounds, such as certain volatile organic compounds and semi-volatile organic compounds. Further, we lack knowledge on which specific chemicals or mixtures of chemicals are associated with the adverse effects, and this is an important area for research.

Limitations of the study include the following: (a) data were based on self-reports, a standard method for survey research with both strengths and shortcomings, (b) all possible products and health effects were not included, although the low percentages for responses in the “other” category indicates the survey captured the primary products and effects, and (c) given the nature of the survey, it was not possible to measure emissions and exposures directly for each respondent.

Results of this study provide compelling evidence that everyday fragranced products can impose serious risks to human health, environmental quality, businesses, and society. Future research can be directed to understanding the types of product ingredients and emissions that are problematic for exposures and health effects, and ways to reduce the impacts. In the meantime, a solution to effectively reduce risks is to avoid, restrict, or reduce the use of fragranced products.

## Electronic supplementary material

ESM 1 (PDF 175 kb)

ESM 2 (PDF 71.5 kb)

## Footnotes

1 The term “fragranced consumer products,” or for brevity “fragranced products,” as used in this article, refers to chemically formulated consumer products with a fragrance (Steinemann 2009). The phrase “fragranced product emissions” refers to emissions from fragranced consumer products. Finally, the words “fragrance(d)” and “scent(ed)” are used interchangeably in this article.
