# Supplementary material for: Fragranced consumer products: exposures and effects from emissions
Source: Air Qual Atmos Health. 2016 Oct 20;9(8):861–6. doi: 10.1007/s11869-016-0442-z (PMC5093181; doi:10.1007/s11869-016-0442-z)
Supplement: Supplementary file 1 — (PDF 175 kb) [file 11869_2016_442_MOESM1_ESM.pdf]

SURVEY RESULTS - USA POPULATION

Which fragranced products are you exposed to, at least once a week, from your own use? Air fresheners and deodorizers (e.g., sprays, solids, oils, disks)

		Frequency	Percent	Valid Percent	Cumulative Percent
Valid	No	309	27.2	27.2	27.2
	Yes	827	72.8	72.8	100.0
	Total	1136	100.0	100.0	

Which fragranced products are you exposed to, at least once a week, from your own use? Personal care products (e.g., soaps, hand sanitizer, lotions, deodorant, sunscreen, shampoos)

		Frequency	Percent	Valid Percent	Cumulative Percent
Valid	No	127	11.2	11.2	11.2
	Yes	1009	88.8	88.8	100.0
	Total	1136	100.0	100.0	

Which fragranced products are you exposed to, at least once a week, from your own use? Cleaning supplies (e.g., all-purpose cleaners, disinfectants, and dishwashing soap)

		Frequency	Percent	Valid Percent	Cumulative Percent
Valid	No	228	20.1	20.1	20.1
	Yes	908	79.9	79.9	100.0
	Total	1136	100.0	100.0	

Which fragranced products are you exposed to, at least once a week, from your own use? Laundry products (e.g., detergents, fabric softeners, dryer sheets)

		Frequency	Percent	Valid Percent	Cumulative Percent
Valid	No	181	15.9	15.9	15.9
	Yes	955	84.1	84.1	100.0
	Total	1136	100.0	100.0	

Which fragranced products are you exposed to, at least once a week, from your own use? Household products (e.g., scented candles, toilet paper, trash bags, baby products)

		Frequency	Percent	Valid Percent	Cumulative Percent
Valid	No	261	23.0	23.0	23.0
	Yes	875	77.0	77.0	100.0
	Total	1136	100.0	100.0	

Which fragranced products are you exposed to, at least once a week, from your own use? Fragrance (e.g., perfume, cologne, after-shave)

		Frequency	Percent	Valid Percent	Cumulative Percent
Valid	No	339	29.8	29.8	29.8
	Yes	797	70.2	70.2	100.0
	Total	1136	100.0	100.0	

Which fragranced products are you exposed to, at least once a week, from your own use? Other

		Frequency	Percent	Valid Percent	Cumulative Percent
Valid	No	1102	97.0	97.0	97.0
	Yes	34	3.0	3.0	100.0
	Total	1136	100.0	100.0	

Which fragranced products are you exposed to, at least once a week, from your own use? None

		Frequency	Percent	Valid Percent	Cumulative Percent
Valid	No	1117	98.3	98.3	98.3
	Yes	19	1.7	1.7	100.0
	Total	1136	100.0	100.0	

Which fragranced products are you exposed to, at least once a week, from others' use? Air fresheners and deodorizers (e.g., sprays, solids, oils, disks)

		Frequency	Percent	Valid Percent	Cumulative Percent
Valid	No	478	42.1	42.1	42.1
	Yes	658	57.9	57.9	100.0
	Total	1136	100.0	100.0	

Which fragranced products are you exposed to, at least once a week, from others' use? Personal care products (e.g., soaps, hand sanitizer, lotions, deodorant, sunscreen, shampoos)

		Frequency	Percent	Valid Percent	Cumulative Percent
Valid	No	385	33.9	33.9	33.9
	Yes	751	66.1	66.1	100.0
	Total	1136	100.0	100.0	

Which fragranced products are you exposed to, at least once a week, from others' use? Cleaning supplies (e.g., all-purpose cleaners, disinfectants, and dishwashing soap)

		Frequency	Percent	Valid Percent	Cumulative Percent
Valid	No	514	45.2	45.2	45.2
	Yes	622	54.8	54.8	100.0
	Total	1136	100.0	100.0	

Which fragranced products are you exposed to, at least once a week, from others' use? Laundry products (e.g., detergents, fabric softeners, dryer sheets)

		Frequency	Percent	Valid Percent	Cumulative Percent
Valid	No	597	52.6	52.6	52.6
	Yes	539	47.4	47.4	100.0
	Total	1136	100.0	100.0	

Which fragranced products are you exposed to, at least once a week, from others' use? Household products (e.g., scented candles, toilet paper, trash bags, baby products)

		Frequency	Percent	Valid Percent	Cumulative Percent
Valid	No	542	47.7	47.7	47.7
	Yes	594	52.3	52.3	100.0
	Total	1136	100.0	100.0	

Which fragranced products are you exposed to, at least once a week, from others' use? Fragrance (e.g., perfume, cologne, after-shave)

		Frequency	Percent	Valid Percent	Cumulative Percent
Valid	No	356	31.3	31.3	31.3
	Yes	780	68.7	68.7	100.0
	Total	1136	100.0	100.0	

Which fragranced products are you exposed to, at least once a week, from others' use? Other

		Frequency	Percent	Valid Percent	Cumulative Percent
Valid	No	1100	96.8	96.8	96.8
	Yes	36	3.2	3.2	100.0
	Total	1136	100.0	100.0	

Which fragranced products are you exposed to, at least once a week, from others' use? None

		Frequency	Percent	Valid Percent	Cumulative Percent
Valid	No	1046	92.1	92.1	92.1
	Yes	90	7.9	7.9	100.0
	Total	1136	100.0	100.0	

Do you experience any health problems when exposed to air fresheners or deodorizers?

		Frequency	Percent	Valid Percent	Cumulative Percent
Valid	Yes	232	20.4	20.4	20.4
	No	791	69.6	69.6	90.1
	Don't know/not sure	108	9.5	9.5	99.6
	Decline to answer	5	.4	.4	100.0
	Total	1136	100.0	100.0	

Which of the following health problems do you experience when exposed to air fresheners or deodorizers? Migraine headaches

		Frequency	Percent	Valid Percent	Cumulative Percent
Valid	No	150	13.2	64.7	64.7
	Yes	82	7.2	35.3	100.0
	Total	232	20.4	100.0	
Missing	System	904	79.6		
Total		1136	100.0		

Which of the following health problems do you experience when exposed to air fresheners or deodorizers? Asthma attacks

		Frequency	Percent	Valid Percent	Cumulative Percent
Valid	No	179	15.8	77.2	77.2
	Yes	53	4.7	22.8	100.0
	Total	232	20.4	100.0	
Missing	System	904	79.6		
Total		1136	100.0		

Which of the following health problems do you experience when exposed to air fresheners or deodorizers? Neurological problems (e.g., dizziness, seizures, head pain, fainting, loss of coordination)

		Frequency	Percent	Valid Percent	Cumulative Percent
Valid	No	196	17.3	84.5	84.5
	Yes	36	3.2	15.5	100.0
	Total	232	20.4	100.0	
Missing	System	904	79.6		
Total		1136	100.0		

Which of the following health problems do you experience when exposed to air fresheners or deodorizers? Respiratory problems (e.g., difficulty breathing, coughing, shortness of breath)

		Frequency	Percent	Valid Percent	Cumulative Percent
Valid	No	124	10.9	53.4	53.4
	Yes	108	9.5	46.6	100.0
	Total	232	20.4	100.0	
Missing	System	904	79.6		
Total		1136	100.0		

Which of the following health problems do you experience when exposed to air fresheners or deodorizers? Skin problems (e.g., rashes, hives, red skin, tingling skin, dermatitis)

		Frequency	Percent	Valid Percent	Cumulative Percent
Valid	No	167	14.7	72.0	72.0
	Yes	65	5.7	28.0	100.0
	Total	232	20.4	100.0	
Missing	System	904	79.6		
Total		1136	100.0		

Which of the following health problems do you experience when exposed to air fresheners or deodorizers? Cognitive problems (e.g., difficulties thinking, concentrating, or remembering)

		Frequency	Percent	Valid Percent	Cumulative Percent
Valid	No	201	17.7	86.6	86.6
	Yes	31	2.7	13.4	100.0
	Total	232	20.4	100.0	
Missing	System	904	79.6		
Total		1136	100.0		

Which of the following health problems do you experience when exposed to air fresheners or deodorizers? Mucosal symptoms (e.g., watery or red eyes, nasal congestion, sneezing)

		Frequency	Percent	Valid Percent	Cumulative Percent
Valid	No	146	12.9	62.9	62.9
	Yes	86	7.6	37.1	100.0
	Total	232	20.4	100.0	
Missing	System	904	79.6		
Total		1136	100.0		

Which of the following health problems do you experience when exposed to air fresheners or deodorizers? Immune system problems (e.g., swollen lymph glands, fever, fatigue)

		Frequency	Percent	Valid Percent	Cumulative Percent
Valid	No	211	18.6	90.9	90.9
	Yes	21	1.8	9.1	100.0
	Total	232	20.4	100.0	
Missing	System	904	79.6		
Total		1136	100.0		

Which of the following health problems do you experience when exposed to air fresheners or deodorizers? Gastrointestinal problems (e.g., nausea, bloating, cramping, diarrhea)

		Frequency	Percent	Valid Percent	Cumulative Percent
Valid	No	201	17.7	86.6	86.6
	Yes	31	2.7	13.4	100.0
	Total	232	20.4	100.0	
Missing	System	904	79.6		
Total		1136	100.0		

Which of the following health problems do you experience when exposed to air fresheners or deodorizers? Cardiovascular problems (e.g., fast or irregular heartbeat, jitteriness, chest discomfort)

		Frequency	Percent	Valid Percent	Cumulative Percent
Valid	No	202	17.8	87.1	87.1
	Yes	30	2.6	12.9	100.0
	Total	232	20.4	100.0	
Missing	System	904	79.6		
Total		1136	100.0		

Which of the following health problems do you experience when exposed to air fresheners or deodorizers? Musculoskeletal problems (e.g., muscle or joint pain, cramps, weakness)

		Frequency	Percent	Valid Percent	Cumulative Percent
Valid	No	205	18.0	88.4	88.4
	Yes	27	2.4	11.6	100.0
	Total	232	20.4	100.0	
Missing	System	904	79.6		
Total		1136	100.0		

Which of the following health problems do you experience when exposed to air fresheners or deodorizers? Other

		Frequency	Percent	Valid Percent	Cumulative Percent
Valid	No	224	19.7	96.6	96.6
	Yes	8	.7	3.4	100.0
	Total	232	20.4	100.0	
Missing	System	904	79.6		
Total		1136	100.0		

Do you experience any health problems from the scent of laundry products coming from a dryer vent?

		Frequency	Percent	Valid Percent	Cumulative Percent
Valid	Yes	142	12.5	12.5	12.5
	No	905	79.7	79.7	92.2
	Don't know/not sure	88	7.7	7.7	99.9
	Decline to answer	1	.1	.1	100.0
	Total	1136	100.0	100.0	

Which of the following health problems do you suffer from the scent of laundry products coming from a dryer vent? Migraine headaches

		Frequency	Percent	Valid Percent	Cumulative Percent
Valid	No	105	9.2	73.9	73.9
	Yes	37	3.3	26.1	100.0
	Total	142	12.5	100.0	
Missing	System	994	87.5		
Total		1136	100.0		

Which of the following health problems do you suffer from the scent of laundry products coming from a dryer vent? Asthma attacks

		Frequency	Percent	Valid Percent	Cumulative Percent
Valid	No	114	10.0	80.3	80.3
	Yes	28	2.5	19.7	100.0
	Total	142	12.5	100.0	
Missing	System	994	87.5		
Total		1136	100.0		

Which of the following health problems do you suffer from the scent of laundry products coming from a dryer vent? Neurological problems (e.g., dizziness, seizures, head pain, fainting, loss of coordination)

		Frequency	Percent	Valid Percent	Cumulative Percent
Valid	No	118	10.4	83.1	83.1
	Yes	24	2.1	16.9	100.0
	Total	142	12.5	100.0	
Missing	System	994	87.5		
Total		1136	100.0		

Which of the following health problems do you suffer from the scent of laundry products coming from a dryer vent? Respiratory problems (e.g., difficulty breathing, coughing, shortness of breath)

		Frequency	Percent	Valid Percent	Cumulative Percent
Valid	No	96	8.5	67.6	67.6
	Yes	46	4.0	32.4	100.0
	Total	142	12.5	100.0	
Missing	System	994	87.5		
Total		1136	100.0		

Which of the following health problems do you suffer from the scent of laundry products coming from a dryer vent? Skin problems (e.g., rashes, hives, red skin, tingling skin, dermatitis)

		Frequency	Percent	Valid Percent	Cumulative Percent
Valid	No	101	8.9	71.1	71.1
	Yes	41	3.6	28.9	100.0
	Total	142	12.5	100.0	
Missing	System	994	87.5		
Total		1136	100.0		

Which of the following health problems do you suffer from the scent of laundry products coming from a dryer vent? Cognitive problems (e.g., difficulties thinking, concentrating, or remembering)

		Frequency	Percent	Valid Percent	Cumulative Percent
Valid	No	127	11.2	89.4	89.4
	Yes	15	1.3	10.6	100.0
	Total	142	12.5	100.0	
Missing	System	994	87.5		
Total		1136	100.0		

Which of the following health problems do you suffer from the scent of laundry products coming from a dryer vent? Mucosal symptoms (e.g., watery or red eyes, nasal congestion, sneezing)

		Frequency	Percent	Valid Percent	Cumulative Percent
Valid	No	94	8.3	66.2	66.2
	Yes	48	4.2	33.8	100.0
	Total	142	12.5	100.0	
Missing	System	994	87.5		
Total		1136	100.0		

Which of the following health problems do you suffer from the scent of laundry products coming from a dryer vent? Immune system problems (e.g., swollen lymph glands, fever, fatigue)

		Frequency	Percent	Valid Percent	Cumulative Percent
Valid	No	123	10.8	86.6	86.6
	Yes	19	1.7	13.4	100.0
	Total	142	12.5	100.0	
Missing	System	994	87.5		
Total		1136	100.0		

Which of the following health problems do you suffer from the scent of laundry products coming from a dryer vent? Gastrointestinal problems (e.g., nausea, bloating, cramping, diarrhea)

		Frequency	Percent	Valid Percent	Cumulative Percent
Valid	No	113	9.9	79.6	79.6
	Yes	29	2.6	20.4	100.0
	Total	142	12.5	100.0	
Missing	System	994	87.5		
Total		1136	100.0		

Which of the following health problems do you suffer from the scent of laundry products coming from a dryer vent? Cardiovascular problems (e.g., fast or irregular heartbeat, jitteriness, chest discomfort)

		Frequency	Percent	Valid Percent	Cumulative Percent
Valid	No	127	11.2	89.4	89.4
	Yes	15	1.3	10.6	100.0
	Total	142	12.5	100.0	
Missing	System	994	87.5		
Total		1136	100.0		

Which of the following health problems do you suffer from the scent of laundry products coming from a dryer vent? Musculoskeletal problems (e.g., muscle or joint pain, cramps, weakness)

		Frequency	Percent	Valid Percent	Cumulative Percent
Valid	No	119	10.5	83.8	83.8
	Yes	23	2.0	16.2	100.0
	Total	142	12.5	100.0	
Missing	System	994	87.5		
Total		1136	100.0		

Which of the following health problems do you suffer from the scent of laundry products coming from a dryer vent? Other

		Frequency	Percent	Valid Percent	Cumulative Percent
Valid	No	138	12.1	97.2	97.2
	Yes	4	.4	2.8	100.0
	Total	142	12.5	100.0	
Missing	System	994	87.5		
Total		1136	100.0		

Do you experience any health problems from being in a room after it has been cleaned with scented products?

		Frequency	Percent	Valid Percent	Cumulative Percent
Valid	Yes	224	19.7	19.7	19.7
	No	838	73.8	73.8	93.5
	Don't know/not sure	73	6.4	6.4	99.9
	Decline to answer	1	.1	.1	100.0
	Total	1136	100.0	100.0	

Which of the following health problems do you experience from being in a room after it has been cleaned with scented? Migraine headaches

		Frequency	Percent	Valid Percent	Cumulative Percent
Valid	No	149	13.1	66.5	66.5
	Yes	75	6.6	33.5	100.0
	Total	224	19.7	100.0	
Missing	System	912	80.3		
Total		1136	100.0		

Which of the following health problems do you experience from being in a room after it has been cleaned with scented? Asthma attacks

		Frequency	Percent	Valid Percent	Cumulative Percent
Valid	No	178	15.7	79.5	79.5
	Yes	46	4.0	20.5	100.0
	Total	224	19.7	100.0	
Missing	System	912	80.3		
Total		1136	100.0		

Which of the following health problems do you experience from being in a room after it has been cleaned with scented? Neurological problems (e.g., dizziness, seizures, head pain, fainting, loss of coordination)

		Frequency	Percent	Valid Percent	Cumulative Percent
Valid	No	177	15.6	79.0	79.0
	Yes	47	4.1	21.0	100.0
	Total	224	19.7	100.0	
Missing	System	912	80.3		
Total		1136	100.0		

Which of the following health problems do you experience from being in a room after it has been cleaned with scented? Respiratory problems (e.g., difficulty breathing, coughing, shortness of breath)

		Frequency	Percent	Valid Percent	Cumulative Percent
Valid	No	115	10.1	51.3	51.3
	Yes	109	9.6	48.7	100.0
	Total	224	19.7	100.0	
Missing	System	912	80.3		
Total		1136	100.0		

Which of the following health problems do you experience from being in a room after it has been cleaned with scented? Skin problems (e.g., rashes, hives, red skin, tingling skin, dermatitis)

		Frequency	Percent	Valid Percent	Cumulative Percent
Valid	No	179	15.8	79.9	79.9
	Yes	45	4.0	20.1	100.0
	Total	224	19.7	100.0	
Missing	System	912	80.3		
Total		1136	100.0		

Which of the following health problems do you experience from being in a room after it has been cleaned with scented? Cognitive problems (e.g., difficulties thinking, concentrating, or remembering)

		Frequency	Percent	Valid Percent	Cumulative Percent
Valid	No	193	17.0	86.2	86.2
	Yes	31	2.7	13.8	100.0
	Total	224	19.7	100.0	
Missing	System	912	80.3		
Total		1136	100.0		

Which of the following health problems do you experience from being in a room after it has been cleaned with scented? Mucosal symptoms (e.g., watery or red eyes, nasal congestion, sneezing)

		Frequency	Percent	Valid Percent	Cumulative Percent
Valid	No	141	12.4	62.9	62.9
	Yes	83	7.3	37.1	100.0
	Total	224	19.7	100.0	
Missing	System	912	80.3		
Total		1136	100.0		

Which of the following health problems do you experience from being in a room after it has been cleaned with scented? Immune system problems (e.g., swollen lymph glands, fever, fatigue)

		Frequency	Percent	Valid Percent	Cumulative Percent
Valid	No	201	17.7	89.7	89.7
	Yes	23	2.0	10.3	100.0
	Total	224	19.7	100.0	
Missing	System	912	80.3		
Total		1136	100.0		

Which of the following health problems do you experience from being in a room after it has been cleaned with scented? Gastrointestinal problems (e.g., nausea, bloating, cramping, diarrhea)

		Frequency	Percent	Valid Percent	Cumulative Percent
Valid	No	192	16.9	85.7	85.7
	Yes	32	2.8	14.3	100.0
	Total	224	19.7	100.0	
Missing	System	912	80.3		
Total		1136	100.0		

Which of the following health problems do you experience from being in a room after it has been cleaned with scented? Cardiovascular problems (e.g., fast or irregular heartbeat, jitteriness, chest discomfort)

		Frequency	Percent	Valid Percent	Cumulative Percent
Valid	No	198	17.4	88.4	88.4
	Yes	26	2.3	11.6	100.0
	Total	224	19.7	100.0	
Missing	System	912	80.3		
Total		1136	100.0		

Which of the following health problems do you experience from being in a room after it has been cleaned with scented? Musculoskeletal problems (e.g., muscle or joint pain, cramps, weakness)

		Frequency	Percent	Valid Percent	Cumulative Percent
Valid	No	201	17.7	89.7	89.7
	Yes	23	2.0	10.3	100.0
	Total	224	19.7	100.0	
Missing	System	912	80.3		
Total		1136	100.0		

Which of the following health problems do you experience from being in a room after it has been cleaned with scented? Other

		Frequency	Percent	Valid Percent	Cumulative Percent
Valid	No	220	19.4	98.2	98.2
	Yes	4	.4	1.8	100.0
	Total	224	19.7	100.0	
Missing	System	912	80.3		
Total		1136	100.0		

Do you experience any health problems from being near someone who is wearing a fragranced product?

		Frequency	Percent	Valid Percent	Cumulative Percent
Valid	Yes	268	23.6	23.6	23.6
	No	798	70.2	70.2	93.8
	Don't know/not sure	68	6.0	6.0	99.8
	Decline to answer	2	.2	.2	100.0
	Total	1136	100.0	100.0	

Which of the following health problems do you experience from being near someone who is wearing a fragranced product? Migraine headaches

		Frequency	Percent	Valid Percent	Cumulative Percent
Valid	No	172	15.1	64.2	64.2
	Yes	96	8.5	35.8	100.0
	Total	268	23.6	100.0	
Missing	System	868	76.4		
Total		1136	100.0		

Which of the following health problems do you experience from being near someone who is wearing a fragranced product? Asthma attacks

		Frequency	Percent	Valid Percent	Cumulative Percent
Valid	No	224	19.7	83.6	83.6
	Yes	44	3.9	16.4	100.0
	Total	268	23.6	100.0	
Missing	System	868	76.4		
Total		1136	100.0		

Which of the following health problems do you experience from being near someone who is wearing a fragranced product? Neurological problems (e.g., dizziness, seizures, head pain, fainting, loss of coordination)

		Frequency	Percent	Valid Percent	Cumulative Percent
Valid	No	227	20.0	84.7	84.7
	Yes	41	3.6	15.3	100.0
	Total	268	23.6	100.0	
Missing	System	868	76.4		
Total		1136	100.0		

Which of the following health problems do you experience from being near someone who is wearing a fragranced product? Respiratory problems (e.g., difficulty breathing, coughing, shortness of breath)

		Frequency	Percent	Valid Percent	Cumulative Percent
Valid	No	150	13.2	56.0	56.0
	Yes	118	10.4	44.0	100.0
	Total	268	23.6	100.0	
Missing	System	868	76.4		
Total		1136	100.0		

Which of the following health problems do you experience from being near someone who is wearing a fragranced product? Skin problems (e.g., rashes, hives, red skin, tingling skin, dermatitis)

		Frequency	Percent	Valid Percent	Cumulative Percent
Valid	No	229	20.2	85.4	85.4
	Yes	39	3.4	14.6	100.0
	Total	268	23.6	100.0	
Missing	System	868	76.4		
Total		1136	100.0		

Which of the following health problems do you experience from being near someone who is wearing a fragranced product? Cognitive problems (e.g., difficulties thinking, concentrating, or remembering)

		Frequency	Percent	Valid Percent	Cumulative Percent
Valid	No	238	21.0	88.8	88.8
	Yes	30	2.6	11.2	100.0
	Total	268	23.6	100.0	
Missing	System	868	76.4		
Total		1136	100.0		

Which of the following health problems do you experience from being near someone who is wearing a fragranced product? Mucosal symptoms (e.g., watery or red eyes, nasal congestion, sneezing)

		Frequency	Percent	Valid Percent	Cumulative Percent
Valid	No	170	15.0	63.4	63.4
	Yes	98	8.6	36.6	100.0
	Total	268	23.6	100.0	
Missing	System	868	76.4		
Total		1136	100.0		

Which of the following health problems do you experience from being near someone who is wearing a fragranced product? Immune system problems (e.g., swollen lymph glands, fever, fatigue)

		Frequency	Percent	Valid Percent	Cumulative Percent
Valid	No	249	21.9	92.9	92.9
	Yes	19	1.7	7.1	100.0
	Total	268	23.6	100.0	
Missing	System	868	76.4		
Total		1136	100.0		

Which of the following health problems do you experience from being near someone who is wearing a fragranced product? Gastrointestinal problems (e.g., nausea, bloating, cramping, diarrhea)

		Frequency	Percent	Valid Percent	Cumulative Percent
Valid	No	237	20.9	88.4	88.4
	Yes	31	2.7	11.6	100.0
	Total	268	23.6	100.0	
Missing	System	868	76.4		
Total		1136	100.0		

Which of the following health problems do you experience from being near someone who is wearing a fragranced product? Cardiovascular problems (e.g., fast or irregular heartbeat, jitteriness, chest discomfort)

		Frequency	Percent	Valid Percent	Cumulative Percent
Valid	No	248	21.8	92.5	92.5
	Yes	20	1.8	7.5	100.0
	Total	268	23.6	100.0	
Missing	System	868	76.4		
Total		1136	100.0		

Which of the following health problems do you experience from being near someone who is wearing a fragranced product? Musculoskeletal problems (e.g., muscle or joint pain, cramps, weakness)

		Frequency	Percent	Valid Percent	Cumulative Percent
Valid	No	251	22.1	93.7	93.7
	Yes	17	1.5	6.3	100.0
	Total	268	23.6	100.0	
Missing	System	868	76.4		
Total		1136	100.0		

Which of the following health problems do you experience from being near someone who is wearing a fragranced product? Other

		Frequency	Percent	Valid Percent	Cumulative Percent
Valid	No	261	23.0	97.4	97.4
	Yes	7	.6	2.6	100.0
	Total	268	23.6	100.0	
Missing	System	868	76.4		
Total		1136	100.0		

**Product Use**	own use	others' use
	1137	1137
	100.00%	100.00%
Yes (Net)	1118	1047
	98.30%	92.10%
Air fresheners and deodorizers	828	658
	72.80%	57.90%
Personal care products	1010	751
	88.80%	66.10%
Cleaning supplies	909	622
	79.90%	54.80%
Laundry products	956	539
	84.10%	47.40%
Household products	876	594
	77.00%	52.30%
Fragrance	798	781
	70.20%	68.70%
Other	34	36
	3.00%	3.20%
None	19	90
	1.70%	7.90%
Total (own use, others' use, or both) = 99.1%		

**Health Effects from Product Exposure**	
	1137
	100.00%
Respiratory problems	211
	18.60%
Mucosal symptoms	184
	16.20%
Migraine headaches	179
	15.70%
Skin problems	121
	10.60%
Asthma attacks	91
	8.00%
Neurological problems	82
	7.20%
Cognitive problems	66
	5.80%
Gastrointestinal problems	63
	5.50%
Cardiovascular problems	50
	4.40%
Immune system problems	45
	4.00%
Musculoskeletal problems	43
	3.80%
Other	19
	1.70%
Total (reporting one or more heath effects)	34.70%

Do any of these health problems substantially limit one or more major life activities, such as seeing, hearing, eating, sleeping, walking, standing, lifting, bending, speaking, breathing, learning, reading, concentrating, thinking, communicating, or working, for you personally?

		Frequency	Percent	Valid Percent	Cumulative Percent
Valid	Yes	195	17.2	49.5	49.5
	No	175	15.4	44.4	93.9
	Don't know/not sure	22	1.9	5.6	99.5
	Decline to answer	2	.2	.5	100.0
	Total	394	34.7	100.0	
Missing	System	742	65.3		
Total		1136	100.0		

Have you ever been unable or reluctant to use the toilets in a public place, because of the presence of an air freshener, deodorizer, or scented product?

		Frequency	Percent	Valid Percent	Cumulative Percent
Valid	Yes	199	17.5	17.5	17.5
	No	896	78.9	78.9	96.4
	Neutral/not sure	40	3.5	3.5	99.9
	Decline to answer	1	.1	.1	100.0
	Total	1136	100.0	100.0	

If you enter a business, and you smell air fresheners or some fragranced product, do you want to leave as quickly as possible?

		Frequency	Percent	Valid Percent	Cumulative Percent
Valid	Yes	229	20.2	20.2	20.2
	No	786	69.2	69.2	89.3
	Neutral/not sure	120	10.6	10.6	99.9
	Decline to answer	1	.1	.1	100.0
	Total	1136	100.0	100.0	

Have you ever been unable or reluctant to wash your hands with soap in a public place, because you know or suspect that the soap is fragranced?

		Frequency	Percent	Valid Percent	Cumulative Percent
Valid	Yes	160	14.1	14.1	14.1
	No	923	81.3	81.3	95.3
	Neutral/not sure	50	4.4	4.4	99.7
	Decline to answer	3	.3	.3	100.0
	Total	1136	100.0	100.0	

Are you aware that a 'fragrance' in a product is typically a chemical mixture of several dozen to several hundred chemicals, mostly synthetic and derived from petrochemicals?

		Frequency	Percent	Valid Percent	Cumulative Percent
Valid	Yes	499	43.9	43.9	43.9
	No	527	46.4	46.4	90.3
	Don't know/not sure	107	9.4	9.4	99.7
	Decline to answer	3	.3	.3	100.0
	Total	1136	100.0	100.0	

Are you aware that fragrance chemicals do not need to be fully disclosed on the product label or material safety data sheet?

		Frequency	Percent	Valid Percent	Cumulative Percent
Valid	Yes	293	25.8	25.8	25.8
	No	734	64.6	64.6	90.4
	Don't know/not sure	107	9.4	9.4	99.8
	Decline to answer	2	.2	.2	100.0
	Total	1136	100.0	100.0	

Are you aware that fragranced products typically emit hazardous air pollutants such as formaldehyde?

		Frequency	Percent	Valid Percent	Cumulative Percent
Valid	Yes	282	24.8	24.8	24.8
	No	764	67.3	67.3	92.1
	Don't know/not sure	86	7.6	7.6	99.6
	Decline to answer	4	.4	.4	100.0
	Total	1136	100.0	100.0	

Are you aware that even so-called natural, green, and organic fragranced products typically emit hazardous air pollutants?

		Frequency	Percent	Valid Percent	Cumulative Percent
Valid	Yes	219	19.3	19.3	19.3
	No	825	72.6	72.6	91.9
	Don't know/not sure	88	7.7	7.7	99.6
	Decline to answer	4	.4	.4	100.0
	Total	1136	100.0	100.0	

If you knew that a fragranced product emitted hazardous air pollutants, would you still use it?

		Frequency	Percent	Valid Percent	Cumulative Percent
Valid	Yes	191	16.8	16.8	16.8
	No	683	60.1	60.1	76.9
	Don't know/not sure	259	22.8	22.8	99.7
	Decline to answer	3	.3	.3	100.0
	Total	1136	100.0	100.0	

Have you ever been prevented from going to some place because you would be exposed to a fragrance product that would make you sick?

		Frequency	Percent	Valid Percent	Cumulative Percent
Valid	Yes	258	22.7	22.7	22.7
	No	818	72.0	72.0	94.7
	Don't know/not sure	58	5.1	5.1	99.8
	Decline to answer	2	.2	.2	100.0
	Total	1136	100.0	100.0	

Has any exposure to fragranced products in your work environment caused you to become sick, lose work days, or lose a job?

		Frequency	Percent	Valid Percent	Cumulative Percent
Valid	Yes	172	15.1	15.1	15.1
	No	909	80.0	80.0	95.2
	Don't know/not sure	54	4.8	4.8	99.9
	Decline to answer	1	.1	.1	100.0
	Total	1136	100.0	100.0	

Would you be supportive of a fragrance-free policy in the workplace?

		Frequency	Percent	Valid Percent	Cumulative Percent
Valid	Yes	604	53.2	53.2	53.2
	No	224	19.7	19.7	72.9
	Neutral/not sure	303	26.7	26.7	99.6
	Decline to answer	5	.4	.4	100.0
	Total	1136	100.0	100.0	

Would you prefer that health care facilities and health care professionals be fragrance-free?

		Frequency	Percent	Valid Percent	Cumulative Percent
Valid	Yes	623	54.8	54.8	54.8
	No	255	22.4	22.4	77.3
	Neutral/not sure	253	22.3	22.3	99.6
	Decline to answer	5	.4	.4	100.0
	Total	1136	100.0	100.0	

If you had a choice between ... flying on an airplane that pumped scented air throughout the passenger cabin, OR flying on an airplane that did not pump scented air throughout the passenger cabin. Which would you choose?

		Frequency	Percent	Valid Percent	Cumulative Percent
Valid	Airplane with scented air	268	23.6	23.6	23.6
	Airplane without scented air	672	59.2	59.2	82.7
	Neutral/not sure	189	16.6	16.6	99.4
	Decline to answer	7	.6	.6	100.0
	Total	1136	100.0	100.0	

If you had a choice between... staying in a hotel with fragranced air OR staying in a hotel without fragranced air. Which would you choose?

		Frequency	Percent	Valid Percent	Cumulative Percent
Valid	Hotel with fragranced air	316	27.8	27.8	27.8
	Hotel without fragranced air	631	55.5	55.5	83.4
	Neutral/not sure	185	16.3	16.3	99.6
	Decline to answer	4	.4	.4	100.0
	Total	1136	100.0	100.0	

What is your household annual income?

		Frequency	Percent	Valid Percent	Cumulative Percent
Valid	Less than $10,000	73	6.4	6.4	6.4
	$10,000 - $49,999	370	32.6	32.6	39.0
	$50,000 - $99,999	371	32.7	32.7	71.7
	$100,000-$149,999	176	15.5	15.5	87.1
	$150,000-$200,000	65	5.7	5.7	92.9
	Over $200,000	28	2.5	2.5	95.3
	Decline to answer	53	4.7	4.7	100.0
	Total	1136	100.0	100.0	

What is your gender?

		Frequency	Percent	Valid Percent	Cumulative Percent
Valid	Male	525	46.2	46.2	46.2
	Female	611	53.8	53.8	100.0
	Total	1136	100.0	100.0	

What is your age?

		Frequency	Percent	Valid Percent	Cumulative Percent
Valid	18-24	125	11.0	11.0	11.0
	25-34	265	23.3	23.3	34.3
	35-44	290	25.5	25.5	59.9
	45-54	252	22.2	22.2	82.0
	55-65	204	18.0	18.0	100.0
	Total	1136	100.0	100.0	
